# Gene Monitoring in Obesity-Induced Metabolic Dysfunction in Rats: Preclinical Data on Breast Neoplasia Initiation

**DOI:** 10.3390/ijms26157296

**Published:** 2025-07-28

**Authors:** Francisco Claro, Joseane Morari, Camila de Angelis, Emerielle Cristine Vanzela, Wandir Antonio Schiozer, Lício Velloso, Luis Otavio Zanatta Sarian

**Affiliations:** 1 Department of Gynecology and Obstetrics, School of Medical Sciences, State University of Campinas (UNICAMP), Campinas 13083-887, Brazil; sarian@unicamp.br; 2 Laboratory of Cell Signaling, Obesity, Comorbidities Research Center, State University of Campinas (UNICAMP), Campinas 13083-887, Brazil; morarij@gmail.com (J.M.); ecv@unicamp.br (E.C.V.); jmorari@unicamp.br (L.V.); 3 Laboratory of Specialized Pathology, School of Medical Sciences, State University of Campinas (UNICAMP), Campinas13083-887, Brazil; camila.angelis@gmail.com; 4 Department of Surgery, Faculty of Medicine of Jundiai (FMJ), Jundiaí 13202-550, Brazil; wandir.s@terra.com.br

**Keywords:** obesity, luminal breast cancer, breast cancer preclinical model, immunocompetent rat model, gene expression, metabolic dysfunction, tumor microenvironment

## Abstract

Obesity and metabolic dysfunction are established risk factors for luminal breast cancer, yet current preclinical models inadequately recapitulate the complex metabolic and immune interactions driving tumorigenesis. To develop and characterize an immunocompetent rat model of luminal breast cancer induced by chronic exposure to a cafeteria diet mimicking Western obesogenic nutrition, female rats were fed a cafeteria diet or standard chow from weaning. Metabolic parameters, plasma biomarkers (including leptin, insulin, IGF-1, adiponectin, and estrone), mammary gland histology, tumor incidence, and gene expression profiles were longitudinally evaluated. Gene expression was assessed by PCR arrays and qPCR. A subgroup underwent dietary reversal to assess the reversibility of molecular alterations. Cafeteria diet induced significant obesity (mean weight 426.76 g vs. 263.09 g controls, *p* < 0.001) and increased leptin levels without altering insulin, IGF-1, or inflammatory markers. Histological analysis showed increased ductal ectasia and benign lesions, with earlier fibroadenoma and luminal carcinoma development in diet-fed rats. Tumors exhibited luminal phenotype, low Ki67, and elevated PAI-1 expression. Gene expression alterations were time point specific and revealed early downregulation of ID1 and COX2, followed by upregulation of MMP2, THBS1, TWIST1, and PAI-1. Short-term dietary reversal normalized several gene expression changes. Overall tumor incidence was modest (~12%), reflecting early tumor-promoting microenvironmental changes rather than aggressive carcinogenesis. This immunocompetent cafeteria diet rat model recapitulates key metabolic, histological, and molecular features of obesity-associated luminal breast cancer and offers a valuable platform for studying early tumorigenic mechanisms and prevention strategies without carcinogen-induced confounders.

## 1. Introduction

Breast cancer (BC) research requires reliable in vivo models to study tumor biology, risk factors, and treatment responses. Although in vitro systems enable controlled investigations, they lack the architectural complexity, heterogeneity, and dynamic interactions present in native tissues, particularly between epithelial and stromal components [1,2]. Immunocompromised mouse models, such as athymic or SCID mice, allow engraftment of human tumors but exclude the influence of the host immune system, which is increasingly recognized as a critical regulator of tumor development, progression, and therapy response [3,4,5].

Immunocompetent rodent models offer an alternative for studying BC within a physiologically intact immune system [6]. However, most such models rely on chemical carcinogens, including DMBA or PhIP, to induce tumorigenesis [4,7]. These agents exert persistent systemic effects, confounding analysis of immune, metabolic, and hormonal pathways [8,9]. Moreover, they do not reflect the endogenous, lifestyle-associated mechanisms—particularly those linked to obesity and metabolic dysfunction—that are increasingly implicated in human luminal BC [10].

An ideal model should reproduce the microenvironment of luminal tumors, induce neoplasms within a feasible time frame, preserve immune integrity, avoid prolonged systemic toxicity, and allow sufficient post-tumor survival for longitudinal assessment. In this context, diet-induced obesity has emerged as a promising strategy. Obesity is known to promote tumorigenesis by altering stromal cell composition, increasing inflammation, and disrupting endocrine signaling [11,12,13,14].

The cafeteria diet, a palatable, energy-dense regimen rich in saturated fats and sugars, mimics Western dietary habits and reliably induces obesity and metabolic syndrome in rodents [15,16]. Obese adipose tissue has been shown to modulate the breast tumor microenvironment through secretion of adipokines, extracellular matrix remodeling, and recruitment of macrophages and fibroblasts with pro-tumorigenic phenotypes [12,17,18]. These mechanisms are particularly relevant to luminal BC, which remains sensitive to hormonal and metabolic modulation [19,20].

In this study, we introduce a novel model of luminal breast cancer in immunocompetent rats based on chronic exposure to the cafeteria diet. We describe the metabolic and histological features of the model, evaluate gene expression changes in mammary tissue, and identify early tumor-promoting alterations preceding overt carcinogenesis. While overall tumor incidence remained low, the systemic and molecular findings support the use of this model to investigate obesity-driven mammary tumorigenesis without the confounding effects of carcinogenic agents.

## 2. Results

### 2.1. Metabolic Analyses

The mean body weight at 26 weeks of age was 263.09 g in the Control group and 426.76 g in the Cafeteria Diet group (*p* < 0.001). The mean Lee Index (LI) values were 298.12 in the Control group and 332.63 in the Cafeteria Diet group (*p* = 0.002). Mean fasting glucose was 76.06 mg/dL (*p* = 0.26), and mean perigonadal fat weight (PFW) was 8.21 g (*p* < 0.001).

To evaluate potential residual effects of the cafeteria diet during reproductive senescence, 30 rats (C3 and D3 groups) were analyzed. The mean body weight in the C3 group was 318.68 g, compared to 382.11 g in the D3 group (*p* = 0.01). The mean LI values were 326.50 and 337.88, respectively (*p* = 0.39). Fasting glucose was 89.43 mg/dL in the Control group and 91.80 mg/dL in the Diet group (*p* = 0.36). PFW and gross fat volume (GFV) remained significantly higher in the Diet group (*p* = 0.046 and *p* = 0.022, respectively; see Table 1 and Table 2).

Comparison of metabolic status showed no significant differences in plasma levels of insulin, insulin-like growth factor 1 (IGF-1), C-peptide, adiponectin, estrone (E1), leukemia inhibitory factor (LIF), or C-reactive protein (CRP) between groups. However, leptin was significantly elevated in the Diet group (mean 8.49 vs. 2.14 in Controls; *p* < 0.001) (Table 3). Estrone (E1) was chosen over estradiol (E2) for analysis due to its stronger association with adipose tissue and its susceptibility to obesity-related alterations.

### 2.2. Histological (Macroscopic and Microscopic Analysis)

Histological analyses revealed that adolescent rats exposed to the cafeteria diet exhibited a significantly higher incidence of ductal ectasia (DE) compared to controls (20% vs. 8%; *p* < 0.001) after just 11 weeks of dietary exposure. However, no significant differences were observed in terminal ductal structures, ductal morphology, or nuclear atypia during young adulthood or reproductive senescence.

**Figure 1:**
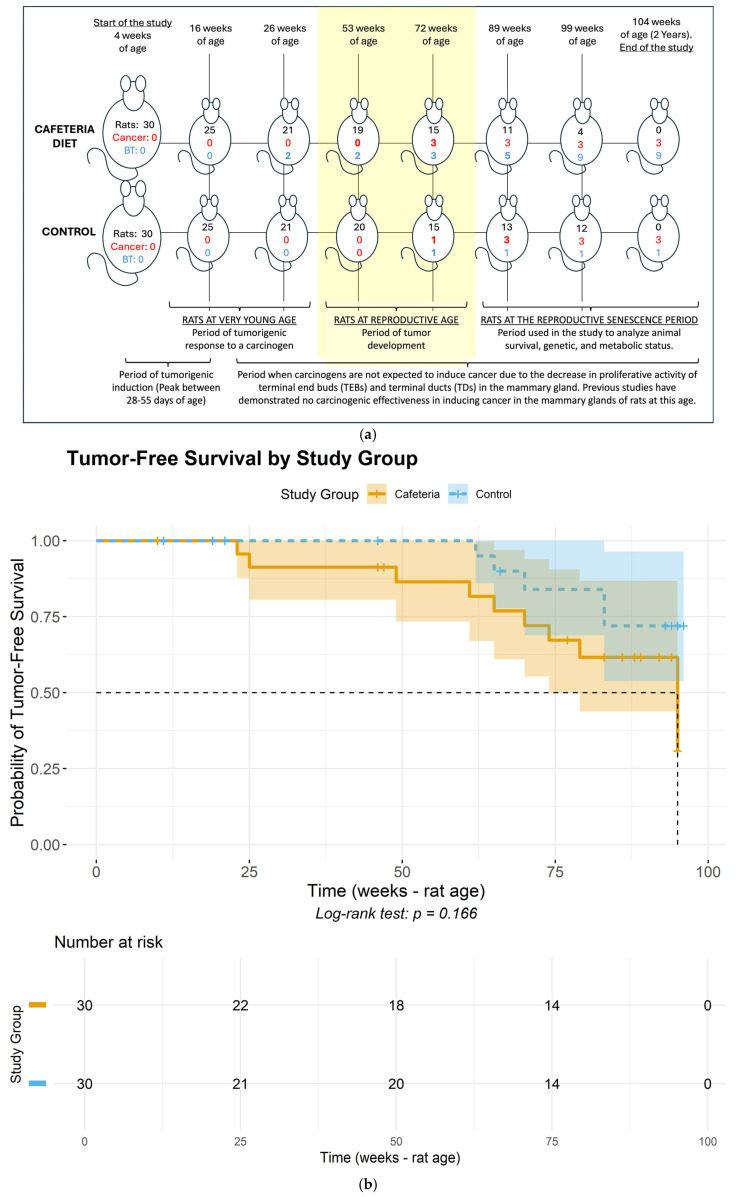

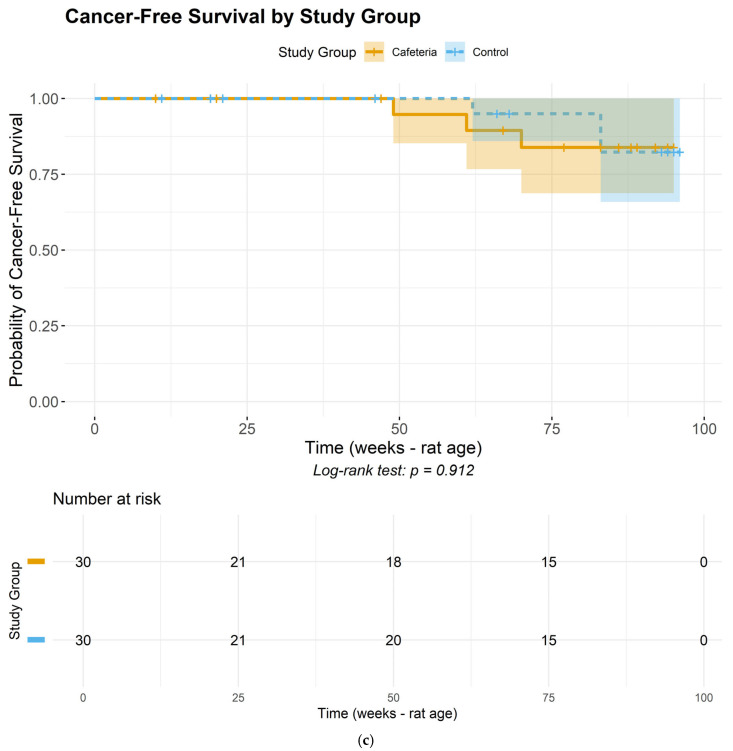
(**a**)**.** Cumulative histopathological findings in the mammary glands throughout the rats’ lifespan. Rats = number of rats alive at each time point; Cancer = cumulative incidence of malignant breast tumors among rats euthanized at each time point; BT = cumulative incidence of benign breast tumors among rats euthanized at each time point. (**b**) Tumor-free (either benign or carcinoma) survival curves for the control and cafeteria groups. (**c**) Cancer-free (carcinoma) survival curves for the control and cafeteria groups. (**a**–**c**) portray the timeline of events regarding tumor development throughout the approximately two-year study duration. Specifically, (**a**) depicts the timeline of tumor development during the study across the cafeteria and Control groups, while (**b**,**c**) depict the tumor-free and cancer-free survival probabilities for the study groups.

**Figure 2:**
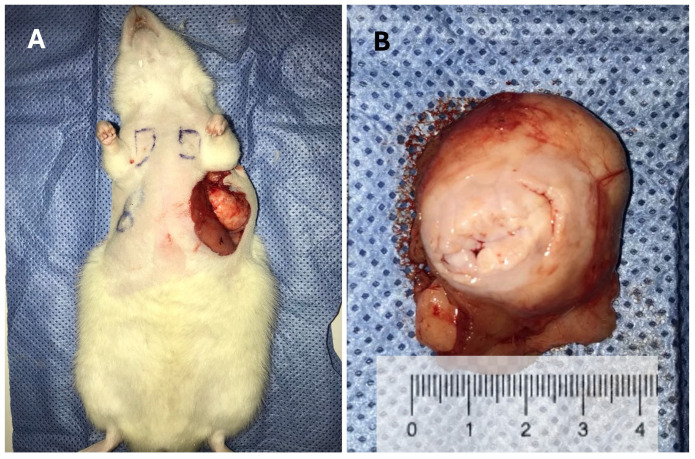
Images of a fibroadenoma in the mammary gland of a very young rat, only 25 weeks old. (**A**) Tumor within the mammary gland. (**B**) Macroscopic view of the fibroadenoma.

**Figure 3:**
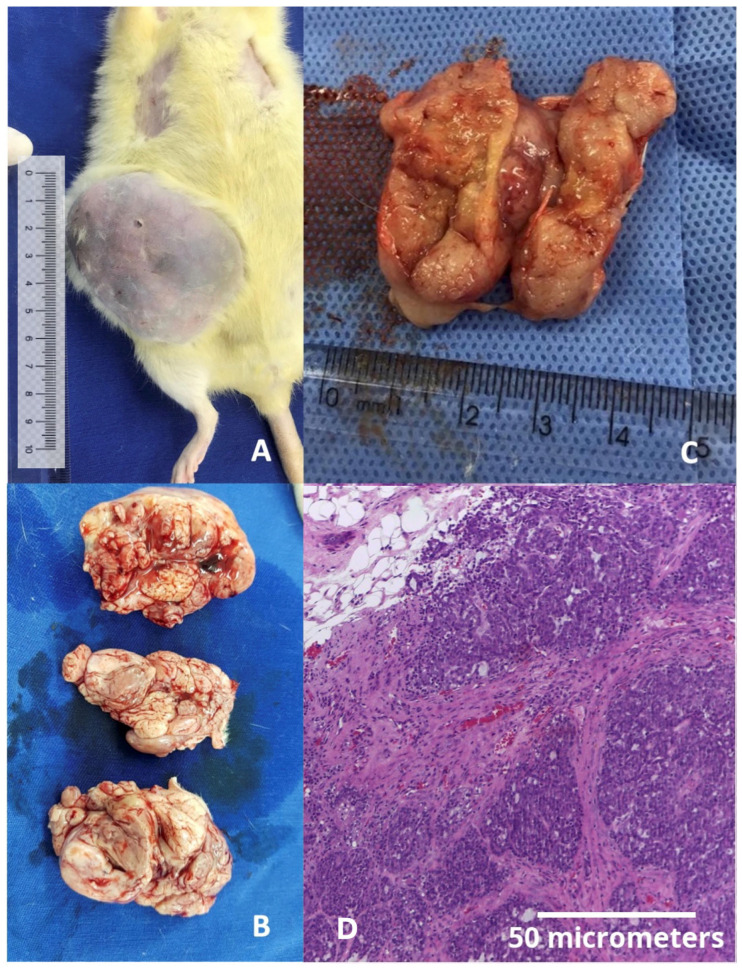
(**A**) Gross image of a fibroadenoma colonized by a ductal carcinoma in a middle-aged rat weighing 577.30 g, taken just before tissue harvesting. This was the largest tumor observed in the study (weighing 53 g) and the only case in which humane endpoint criteria were applied, as the tumor approached 10% of the animal’s body weight seven weeks after initial detection. Notably, this size was reached under rigorous daily monitoring, with no signs of weight loss, ulceration (as shown), pain, distress, impaired mobility, or behavioral changes, in accordance with institutional ethical guidelines. (**B**) Macroscopic view of the same fibroadenoma shown in panel (**A**). (**C**) Macroscopic view of a ductal carcinoma in a middle-aged rat. (**D**) Microscopic view of a ductal carcinoma stained with hematoxylin and eosin (H&E), 400× magnification.

Regarding tumor onset, the Cafeteria Diet group had two fibroadenomas develop within the first 20 weeks of exposure to the cafeteria diet (Figure 1 and Figure 2), while the Control group had the first fibroadenoma appear only in rats at 69 weeks of age. At 72 weeks of age (reproductive age), six rats in the Cafeteria Diet group developed tumors, including three fibroadenomas and three ductal carcinomas (developed inside the fibroadenoma), Figure 3. In the Control group, only two rats had tumors, one fibroadenoma and one ductal carcinoma colonizing a fibroma. This represents a tumor incidence of 24% in the Cafeteria Diet group compared to 8% in the Control group. The incidence of cancer alone was three times higher in the Cafeteria Diet group. The survival analyses showed no tumor-free (log rank *p* = 0.17) nor survival cancer-free (log-rank *p* = 0.91) probability differences for the control and cafeteria groups.

**Figure 4:**
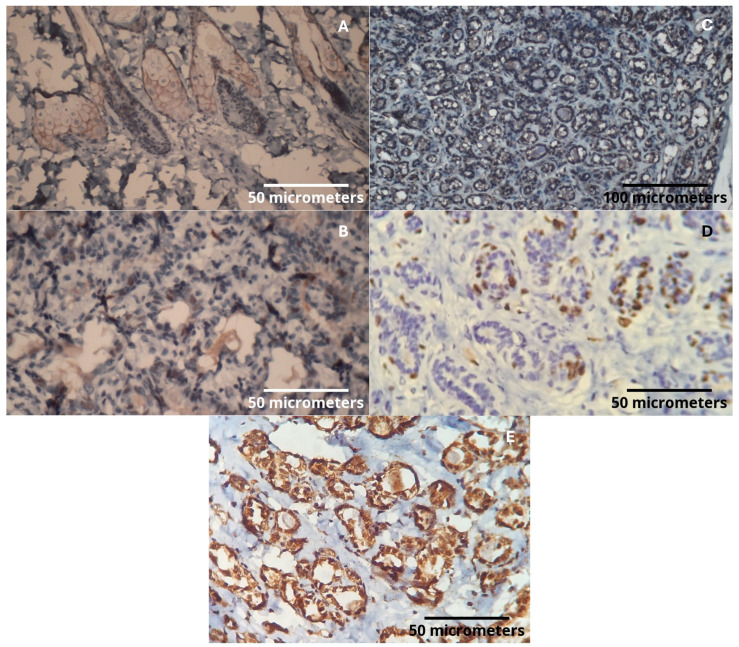
(**A**) HER2: Positive expression in sebaceous glands (internal positive control), (400× magnification). (**B**) Estrogen receptor: Nuclear positivity in the tumor (400× magnification). (**C**) Progesterone receptor: Under dilution adjustment. Reaction with background in the epithelium, making nuclear expression evaluation not possible (200× magnification). (**D**) Ki67: Cellular proliferation index with nuclear marking (400× magnification). (**E**) PAI-1: Intense cytoplasmic expression in tumor epithelial cells (400× magnification).

By approximately 85 weeks of age, during reproductive senescence, five rats in the Cafeteria Diet group developed tumors (three fibroadenomas, two intraductal papillomas), whereas two ductal carcinomas were observed in the Control group. Detailed histopathological findings are shown in Tables S1 and S2. Immunohistochemistry revealed that tumors from the Cafeteria Diet group displayed luminal phenotype, were HER2-negative, had low Ki67 (<1%), and showed strong PAI-1 expression, mirroring the profile of luminal breast cancers in humans (Figure 4).

### 2.3. PCR Array Analysis

(A) Comparison: Control group (n = 30 breasts) vs. Cafeteria Diet at 16 weeks (n = 30 breasts).
  - Upregulated genes (fold change > 2): Muc1, Twist1.
  - Downregulated genes (fold change < 0.5): Abcg2, Ccnd2.
(B) Comparison: Cancer-free Control group (n = 21 breasts) vs. Cancer-free breasts from tumor-bearing Cafeteria Diet rats (n = 15 breasts) at 72 weeks.
  - Upregulated genes: Abcg2, Bad, Bcl2, Birc5, Brca1, Cdh1, Cdkn1a, Csf1, Cst6, Egfr, Gata3, Gli1, Gstp1, Hic1, Igf1, Igfbp3, Il6, Krt18, Mmp2, Muc1, Myc, Notch1, Plau, Sfn, Tgfb1, Twist1, Vegfa, and Xbp1.
  - Downregulated genes: Abcb1a, Ccnd1, Cdh13, Ctnnb1, Erbb2, and Id1.
(C) Comparison: Cancer-free Control group (n = 21 breasts) vs. Cancerous breasts from Cafeteria Diet rats (n = 3 breasts) at 72 weeks.
  - Upregulated genes: Brca2, Ccnd2, Cdh1, Egfr, Gata3, Krt5, Muc1, Notch1, Sfrp1, Twist1, and Vegfa.
  - Downregulated genes: Abcb1a, Adam23, Atm, Bad, Ccnd1, Ccne1, Cdk2, Cdkn1c, Ctnnb1, Egf, Foxa1, Gli1, Igf1, Krt19, Mapk3, Mapk8, Mgmt1, Mlh1, Mmp9, Nr3c1, Pgr, Plau, Prdm2, Pten, Ptgs2, Rarb, Rb1, Slit2, Snai2, and Tp73.

### 2.4. qPCR Validation and Expression Dynamics

To validate PCR array findings, we performed qPCR using distinct probes (Table 4) to assess expression in mammary tissues and tumors across both groups. Gene expression of mammary tissue and tumors in both the control and Cafeteria Diet groups for these specified genes was examined.

**Figure 5:**
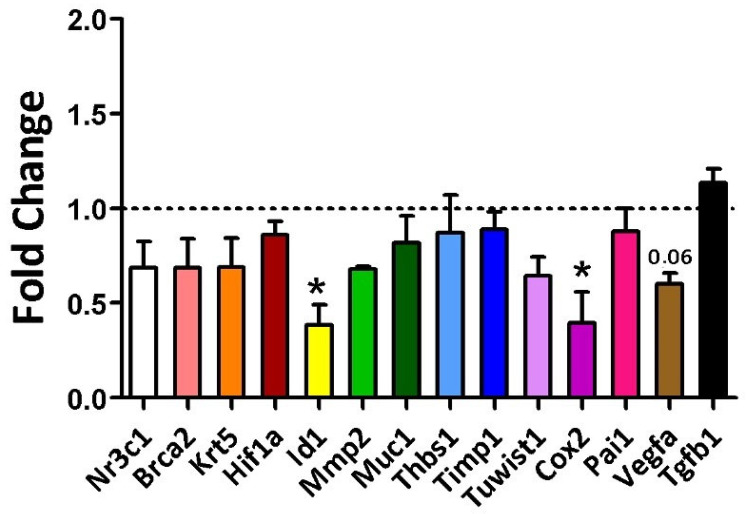
Gene expression of the evaluated markers in 16-week-old rats was analyzed using Student’s *t*-test for the Control group (regular rodent chow, n = 30 breasts) versus the Cafeteria diet (n = 30 breasts). * = *p*-value < 0.05. ID1 and Cox2 showed statistically significant fold changes, with *p*-values of 0.021 and 0.014, respectively. The *p*-value for VEGFA (vascular endothelial growth factor A) was 0.06.

At 16 weeks of age, an initial evaluation of the sample’s homogeneity was conducted in the rats. Figure 1 illustrates the number of animals included in each of the analyses described below. A non-normal distribution was identified for KRT5 (*p* = 0.04) and MMP-2 (*p* = 0.02), whereas all other variables exhibited a normal distribution, indicating data consistency within each group. During this period, gene expression differences between the groups were observed for Cox2 and Id1 markers. The average fold change for Cox2 was 0.40 in the diet group compared to the control (*p* = 0.014), and for Id1, it was 0.70 (Diet, *p* = 0.021) (Figure 5).

**Figure 6:**
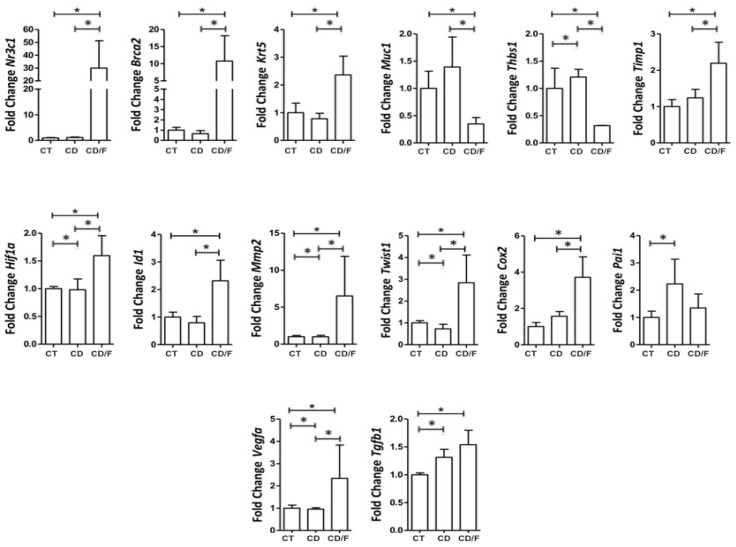
Gene expression of breast tumor markers in 25-week-old rats was compared among the following groups: a Control group of healthy rats (CT; n = 24 breasts), a group of rats fed a cafeteria diet (CD; n = 22 breasts), and a group of rats with fibroadenoma fed a cafeteria diet (CD/F; n = 2 breasts). Although the fibroadenoma group had only two samples, the standard deviation values derived from such a limited sample size are not statistically meaningful. Nevertheless, the values observed were markedly different from those of healthy breast tissue. * = *p*-value < 0.05.

At 25 weeks, corresponding to fibroadenoma emergence, the Diet group showed increased expression of MMP2 (*p* = 0.036), THBS1 (*p* = 0.03), TWIST1 (*p* = 0.015), and PAI-1 (*p* < 0.001). Markers of proliferation, such as HIF1α, VEGFα, and TGF-β, were also significantly elevated (*p* < 0.001). Notably, fibroadenomas showed significantly elevated expression of all tumor markers compared to tumor-free breasts in both groups (Figure 6).

**Figure 7:**
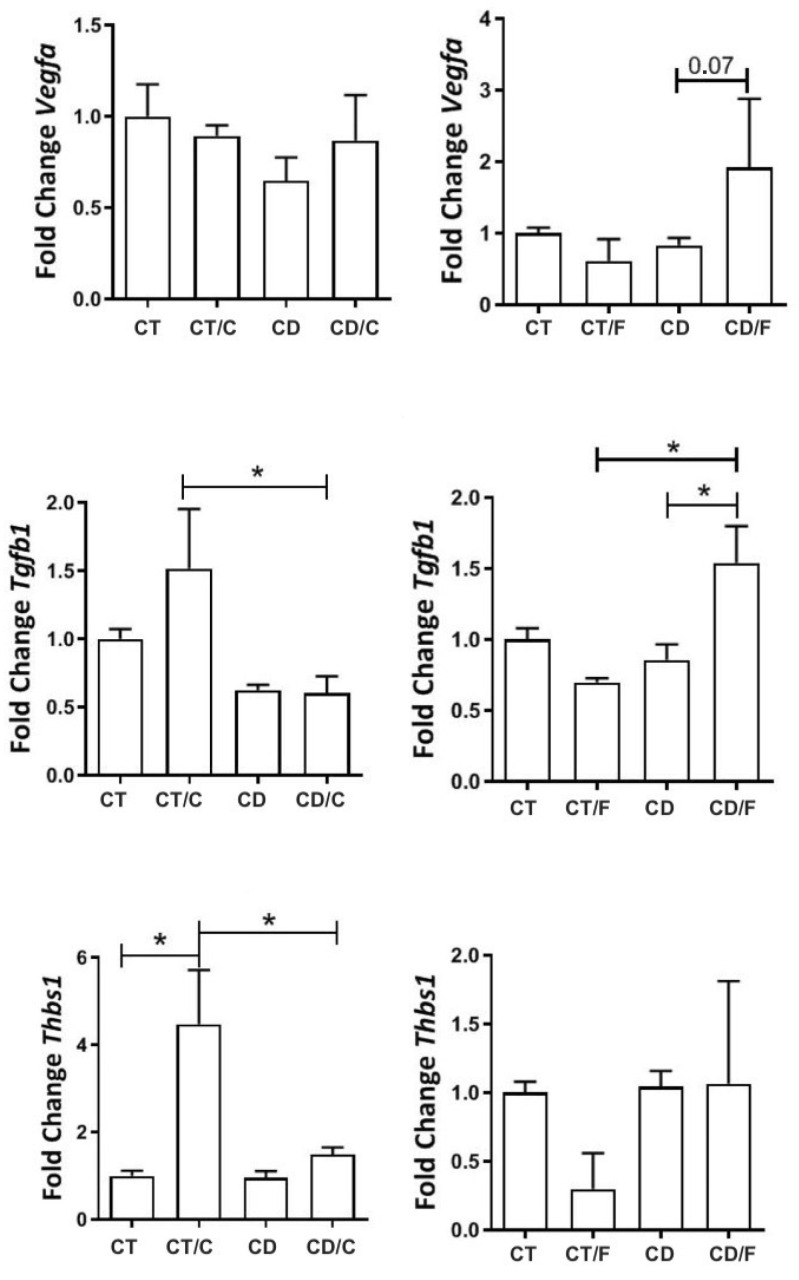
Gene expression of proliferative markers in rats aged 72 to 89 weeks, comparing the left column, a Control group with healthy rats (CT, n = 21 breasts), a diet Control group with rats that developed breast cancer (CT/C, n = 4 breasts), a group of healthy rats fed a cafeteria diet (CD, n = 27 breasts), and a group of rats with breast cancer fed a cafeteria diet (CD-C, n = 3 breasts). In the right column, a Control group with healthy rats (CT, n = 21 breasts), a diet Control group with rats that developed fibroadenoma (CT/F, n = 3 breasts), a group of healthy rats fed a cafeteria diet (CD, n = 27 breasts), and a group of rats with fibroadenoma fed a cafeteria diet (CD/F, n = 3 breasts). * = *p*-value < 0.05.

Between 72–89 weeks, we analyzed gene expression by functional categories (proliferation, inflammation, repair). VEGFα expression was comparable between groups but tended to be higher in fibroadenomas from the Diet group (*p* = 0.07). THBS1 was significantly higher in breast cancer samples from Control rats. TGF-β1 was elevated in all tumors compared to normal tissues, regardless of group (Figure 7).

**Figure 8:**
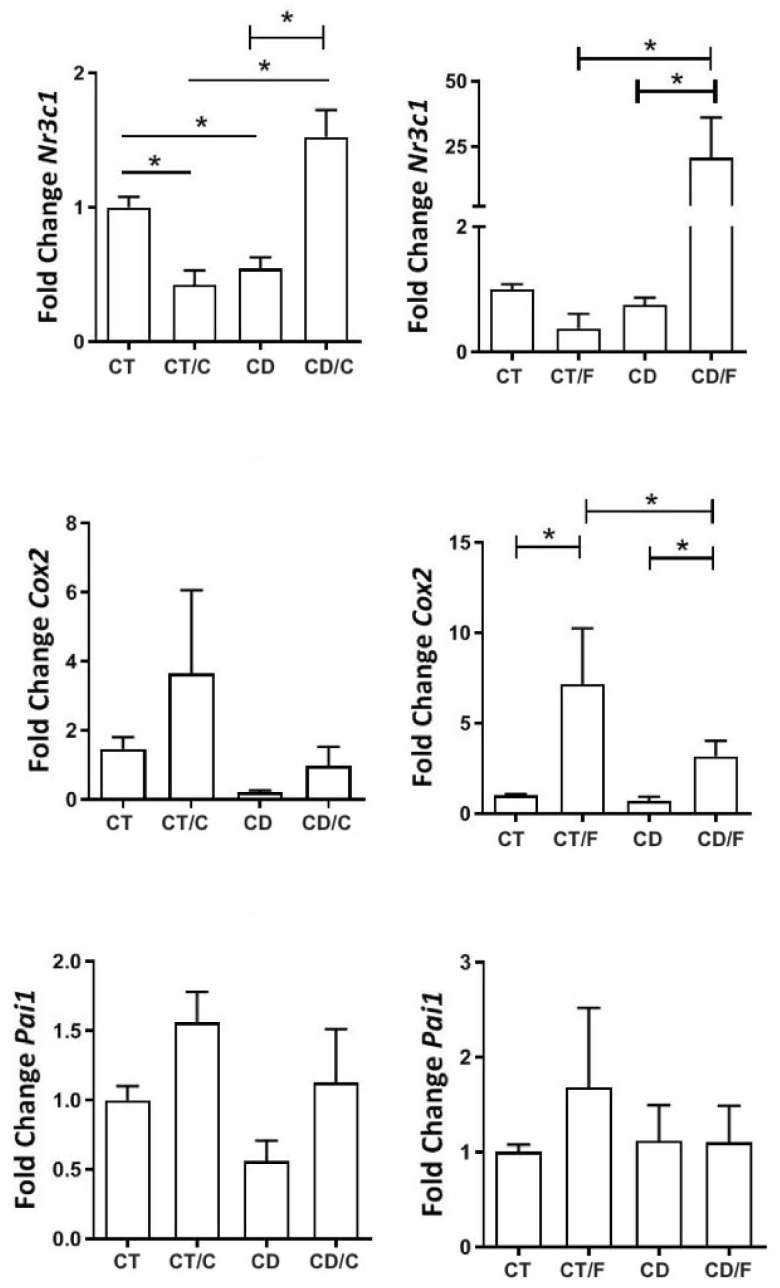
Gene expression of inflammatory markers in rats aged 72 to 89 weeks, comparing in the left column, a Control group with healthy rats (CT, n = 21 breasts), a diet Control group with rats that developed breast cancer (CT/C, n = 4 breasts), a group of healthy rats fed a cafeteria diet (CD, n = 27 breasts), and a group of rats with breast cancer fed a cafeteria diet (CD-C, n = 4 breasts). In the right column, a Control group with healthy rats (CT, n = 21 breasts), a diet Control group with rats that developed fibroadenoma (CT/F, n = 3 breasts), a group of healthy rats fed a cafeteria diet (CD, n = 27 breasts), and a group of rats with fibroadenoma fed a cafeteria diet (CD/F, n = 3 breasts). * = *p*-value < 0.05.

Among inflammatory markers, NR3C1 showed lower expression in healthy mammary tissue from the Diet group but was significantly higher in fibroadenomas and lower in cancers from the Control group. COX-2 was elevated only in fibroadenomas. PAI-1 expression remained similar across groups (Figure 8).

**Figure 9:**
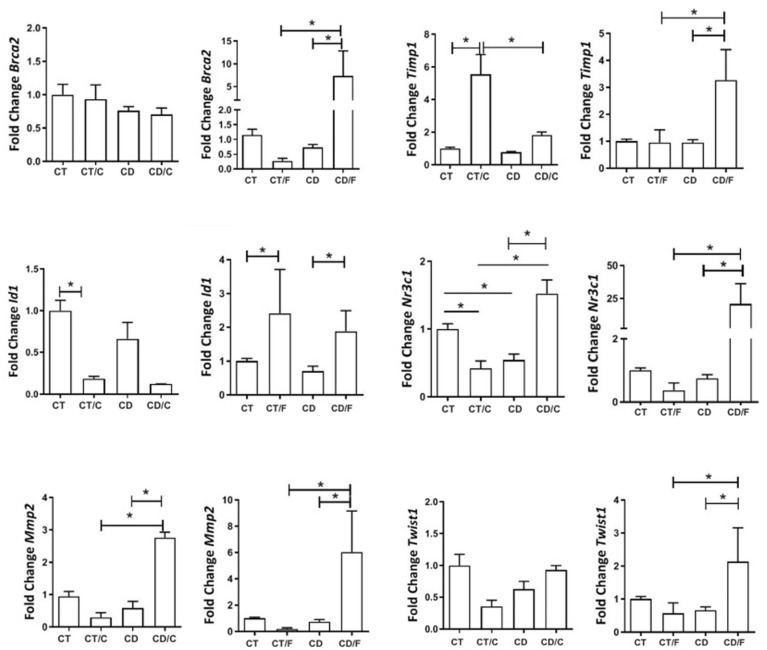
Gene expression of repair markers in rats aged 72 to 89 weeks, comparing a Control group with healthy rats (CT, n = 21 breasts), a diet Control group with rats that developed breast cancer (CT/C, n = 4 breasts), a group of healthy rats fed a cafeteria diet (CD, n = 27 breasts), and a group of rats with breast cancer fed a cafeteria diet (CD/C, n = 3 breasts). It was also compared a Control group with healthy rats (CT, n = 21 breasts), a diet Control group with rats that developed fibroadenoma (CT/F, n = 3 breasts), a group of healthy rats fed a cafeteria diet (CD, n = 27 breasts), and a group of rats with fibroadenoma fed a cafeteria diet (CD/F, n = 3 breasts). * = *p*-value < 0.05.

Among the markers associated with cell repair, BRCA2 displayed significantly higher expression in fibroadenoma samples from rats exposed to the diet. Both ID1 (an MMP2 inhibitor) and MMP2 exhibited a significant decrease in expression in breast cancer cases within the Control group, coupled with greater expression in fibroadenoma samples. MUC1 exhibited higher expression in healthy breasts within the diet group compared to the Control group. In contrast, it was significantly lower among fibroadenoma samples in rats exposed to the cafeteria diet. TIMP1 displayed significant expression among cancer samples in the Control group and healthy breasts (regardless of group), as well as between fibroadenoma cases in the diet group and healthy breasts (independent of the group). TWIST1 exhibited significantly higher expression in fibroadenomas within the group exposed to the cafeteria diet. KRT5 and HIF1-alpha were expressed similarly across all groups (Figure 9).

**Figure 10:**
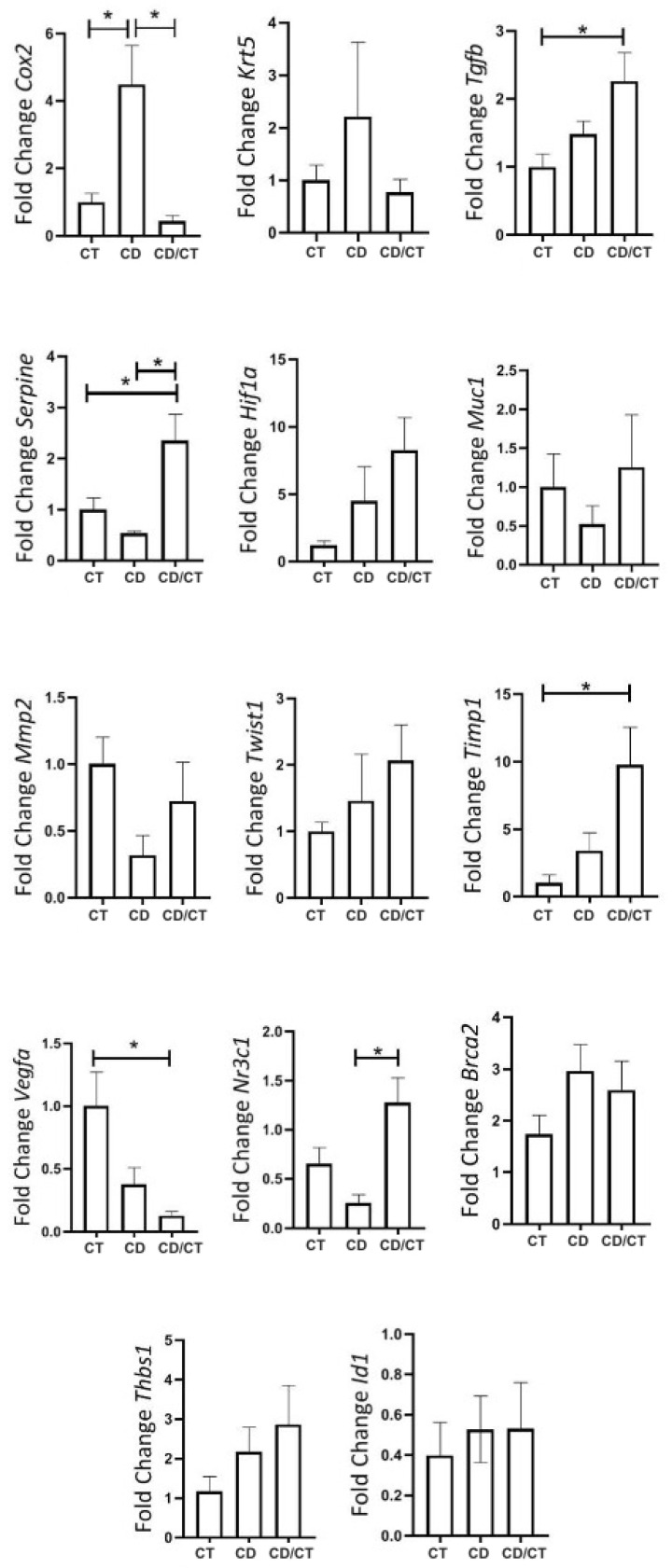
Gene expression of tumor markers in rats with reproductive senescence (102 weeks of age), comparison between samples of healthy mammary glands exposed to different diets. Regular rodent chow was used as the control (CT, n = 30 breasts), cafeteria diet (CD, n = 6 breasts) and rats that received a cafeteria diet from their fourth week of life until 98 weeks of age, with their diets replaced by regular rodent chow in the last 4 weeks of life (CD/CT, n = 14 breasts). * = *p*-value < 0.05.

During reproductive senescence, only benign tumors were observed in the Diet group, while two ductal carcinomas appeared in the Control group. To investigate the potential reversibility of diet-induced effects, we conducted a dietary reversal (cafeteria to standard chow) for 4 weeks. This intervention normalized COX-2, increased TIMP1 and TGF-β, and reduced VEGFα and PAI-1 expression (Figure 10).

**Figure 11:**
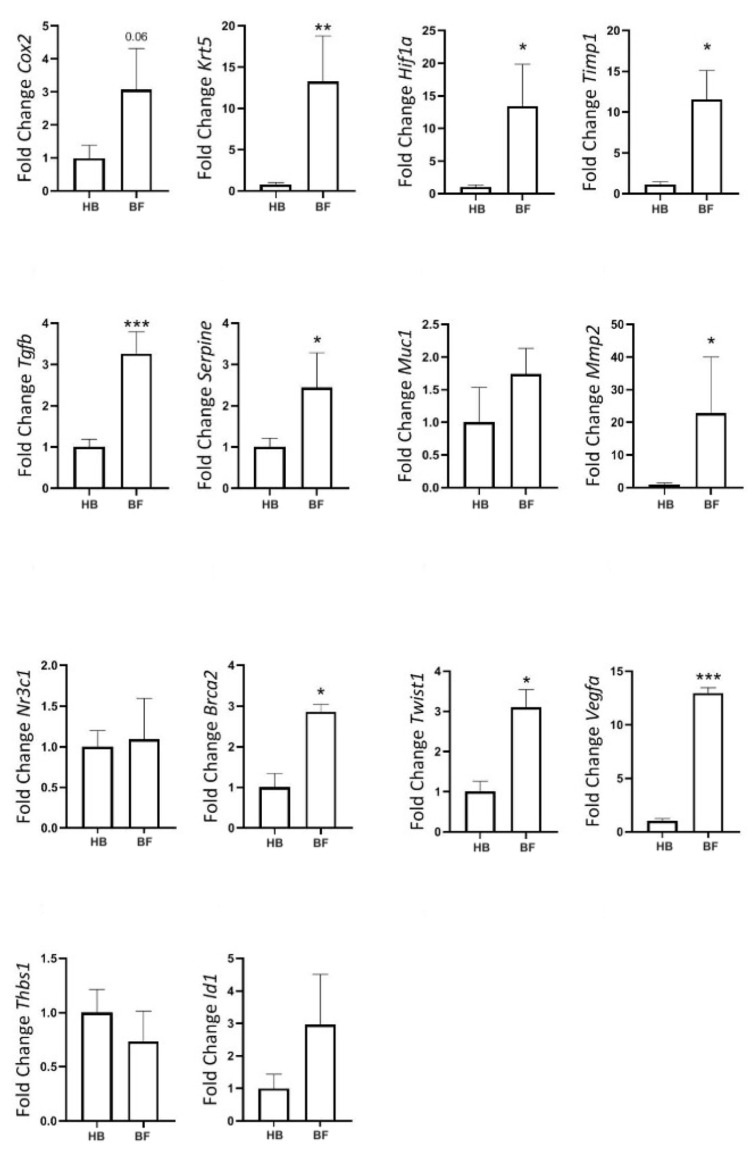
Gene expression of tumor markers in rats at the age of reproductive senescence (at 102 weeks of age) that had mammary fibroadenomas and received a cafeteria diet from their fourth week of life until 98 weeks of age, with their diets replaced by regular rodent chow in the last 4 weeks of life. Comparison between healthy mammary glands (HB, n = 14 breasts) and mammary glands with fibroadenomas (BF, n = 4 breasts). * = *p*-value < 0.05. ** = *p*-value < 0.01. *** = *p*-value < 0.001.

Finally, we compared gene expression between fibroadenomas and healthy tissue in elderly rats. KRT5, TGF-β, PAI-1, HIF-1α, TIMP1, MMP2, TWIST1, VEGFα, and BRCA2 were all significantly upregulated in fibroadenomas (Figure 11). 

## 3. Discussion

Our findings demonstrate that the cafeteria diet induces significant biological changes in the mammary gland of young rats, promoting a metabolically altered and tumor-permissive microenvironment. These results are consistent with the literature, indicating that obesity and high-fat diets contribute to mammary carcinogenesis through endocrine, inflammatory, and stromal pathways [4,5,12,21,22,23,24,25,26,27]. Although the model produced substantial metabolic and transcriptional changes associated with early tumor promotion, the observed incidence of malignant transformation was relatively low. This highlights the model’s relevance for studying early microenvironmental alterations rather than overt tumorigenesis.

Histopathological analysis revealed an increased incidence of ductal ectasia and benign lesions, including fibroadenomas, in cafeteria-fed rats. Additionally, early gene expression alterations, such as downregulation of ID1 and COX2 at 16 weeks, suggest an impact of dietary fat on inflammatory and differentiation-related pathways. These findings align with previous studies linking metabolic dysfunction with altered mammary development and cancer susceptibility [28,29,30].

Notably, the timing of tumor onset was earlier in cafeteria-fed animals compared to controls, with cases of fibroadenoma and luminal carcinoma arising earlier in life. This observation mirrors human epidemiological data that associate early-life obesity with increased breast cancer risk [21,22,23]. Although the overall lifetime incidence of carcinoma was similar across groups, this temporal shift reinforces the idea that metabolic disruptions can accelerate tumorigenesis under permissive conditions.

An important advantage of this model is the reversibility of its metabolic effects. Gene expression profiles normalized following brief dietary withdrawal, allowing potential assessment of therapeutic or preventive interventions without the confounding effects of persistent systemic toxicity, commonly seen with chemical carcinogens [7,24,25,26]. However, in older rats, chronic exposure to the cafeteria diet resulted in increased mortality—likely from cardiometabolic complications and infection—rather than tumor burden per se.

Gene expression analysis showed a biphasic inflammatory response: early suppression of inflammatory mediators was followed by later upregulation of genes involved in inflammation, proliferation, and extracellular matrix remodeling, such as MMP2, THBS1, Twist1, and PAI1. These markers have been associated with breast cancer development in both experimental and clinical studies [21,27,31,32,33]. The pattern suggests a progressive remodeling of the breast stroma in response to chronic metabolic stress [1,2,3,6,9,11,13,14].

It is important to clarify that the observed molecular changes are time-dependent. The gene expression findings suggest that the cafeteria diet induced an earlier onset of cellular proliferation and inflammatory responses compared to the Control group. However, this does not imply that the expression of specific markers remains constant over time. It is well established that this rat strain has a predisposition to developing spontaneous mammary tumors, particularly with advancing age, as also evidenced in our study [17,29,30,34]. Therefore, it is plausible that the differences observed at 16 weeks were no longer apparent at 25 weeks due to age-related alterations that naturally occur in the mammary tissue of control animals. In other words, the diet appeared to accelerate biological changes that would otherwise manifest later, around 25 weeks of age. This interpretation is supported by our histological analyses, which revealed earlier mammary alterations and tumor development in younger animals from the diet group compared to controls. The similarity in marker expression between the diet and Control groups at 25 weeks does not diminish the relevance of the early differences. Rather, it suggests that at a certain point, the inflammatory and proliferative responses converge, likely as a result of aging in both groups. The PCR results were consistent with the early onset of tumor development—including the identification of two fibroadenomas in very young rats—and with tumor types typically observed in reproductively active females. In addition, we included positive PCR controls derived from fibroadenomas and carcinomas, which further support the specificity and reliability of our molecular findings.

Despite its strengths, this model has notable limitations. The low incidence of malignant tumors (~12%) and high frequency of benign lesions (~50%) necessitate large cohorts for statistically powered studies focused on cancer endpoints. As such, the model is particularly suitable for investigating early-stage tumorigenesis, tissue remodeling, and the interaction between diet, adipose tissue, and the immune microenvironment [4,8,10,15,16,17].

To increase tumor yield while preserving physiological relevance, future refinements may involve combining the cafeteria diet with low-dose carcinogens, which have previously been shown to enhance tumorigenesis without systemic toxicity [21,24]. Environmental modifiers—such as altered light cycles, chronic stress, or low-dose ethanol—may also enhance tumor development in metabolically primed tissues [22,23,24,35,36].

In conclusion, the cafeteria diet model offers a physiologically relevant, immunocompetent platform for studying obesity-driven alterations in mammary tissue and their contribution to breast cancer initiation. Although limited by a low malignant transformation rate, its reproducibility, reversibility, and translational fidelity make it a valuable tool for dissecting the early events linking metabolic dysfunction and breast cancer pathogenesis.

## 4. Materials and Methods

### 4.1. Cases 

Sixty female Sprague–Dawley rats (Rattus norvegicus) aged 4 weeks and born on the same day were used. The choice of rat breed and age was based on previous studies highlighting their susceptibility to breast cancer development when exposed to risk factors [17,18,19,20,29,30,34,37]. Hypercaloric diet or obesity has been reported as a potential inducer of breast cancer in rats [27,33,36,38,39,40,41,42,43,44,45,46]. The study adhered to all ARRIVE (Animal Research: Reporting of In Vivo Experiments) [47] and NRC (US, 2011) [48] guidelines and received approval from the institutional Ethics Committee for the Use of Animals under protocol 148/2017. The animals were housed at the Jundiaí School of Medicine’s animal facility in cages of two or three, maintained under 12 h light cycles, with constant temperature, and ad libitum access to food and water. Daily care and cage cleaning were provided by research staff who were trained in animal care/handling.

The rats were randomly divided into two groups: Control and Cafeteria Diet. The animals in the Control group received a standard diet consisting of regular chow for rodents (BioBase^®^, Águas Frias, Brazil), which contained 3.8 kcal/g (70% carbohydrate, 20% protein, and 10% fat), along with access to water ad libitum. The animals in the cafeteria group received a cafeteria diet, adapted from Goularte et al. (2012) [49], consisting of standard chow for rodents (BioBase^®^, Brazil), Italian salami (Sadia^®^, Concórdia, Brazil), bread (Nutrella^®^, Sao Paulo, Brazil), cheese snack balls (Cheetos, Pepsico^®^, Sao Paulo, Brazil), marshmallows (Fini^®^, Jundiaí, Brazil), mixed sausages (Sadia^®^, Brazil), chocolate cake (Renata, Selmi^®^, Sumaré, Brazil), cornflour biscuits (Zadimel^®^, Marechal Cândido Rondon, Brazil), mortadella (Frimesa^®^, Medianeira, Brazil), bacon-flavored snacks (Troféu, Santa Helena^®^, Ribeirão Preto, Brazil), chocolate wafers (Bauducco^®^, Guarulhos, Brazil), and 350 mL of degassed Cola-Cola (Coca-Cola^®^, Rio de Janeiro, Brazil) or Guaraná (Antarctica, AmBev^®^, Sao Paulo, Brazil), alternated weekly. 

This diet provided 5.4 kcal/g (Table 5). The food items were changed daily and offered in pre-established combinations (diets 1, 2, and 3) as follows:

- Diet 01: Standard chow for rodents 15 g, Salami 10 g, Bread 02 units, Cheese snack balls 18 units, Marshmallow 01 unit.
- Diet 02: Standard chow for rodents 15 g, Sausage 20 g, Chocolate Cake 22 g, Cornflour biscuits 03 units, Marshmallow 01 unit.
- Diet 03: Standard chow for rodents 15 g, Mortadella 10 g, Bacon chips 15 g, Chocolate wafers 03 units, Marshmallow 01 unit.

The rats were euthanized according to the study schedule based on the following criteria: 5 rats from each group (a total of 10 rats) at 16 weeks of age, and 4 rats from each group at 25 weeks of age (totaling 8 rats at this period). From then on, they were euthanized 8 weeks after tumor identification or by humane endpoint criteria according to NRC (US, 2011) [48] and CONCEA (Brazil, 2016–RN 33) [50], always accompanied by a Control group rat or when any signs of distress were observed. Finally, all remaining rats were euthanized at the end of the study, at 2 years of age. The rats were euthanized at these predefined times with a lethal dose of sodium pentobarbital administered via intraperitoneal injection, with tissue extraction performed following confirmation of death.

### 4.2. Murinometric Data

**Figure 12:**
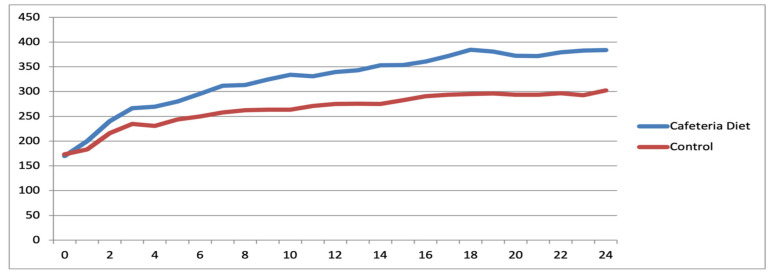
Evolutionary trajectory of mean animal weights within the Diet and Control groups among reproductive-age rats.

**Figure 13:**
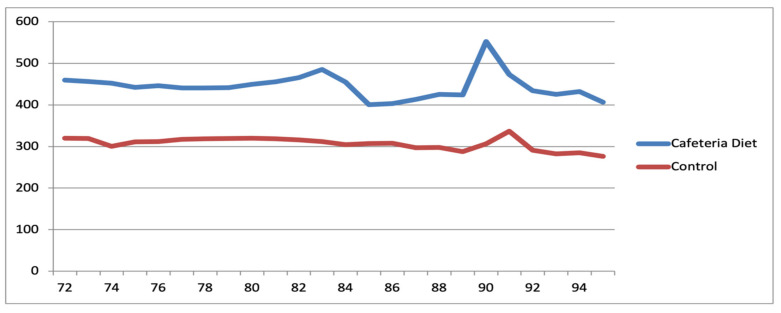
Evolutionary trajectory of mean animal weights within the Diet and Control groups among rats after reproductive senescence.

The weight of the rats was collected weekly, and an individual growth curve was plotted to monitor the progress of each animal (Figure 12 and Figure 13). Weekly palpation of the 6 thoracic breasts and their extensions (axillary and abdomino-inguinal) was performed, and the data were recorded. When a tumor was identified, its size and characteristics were evaluated weekly. Naso-anal length measurement was conducted only at the time of rat euthanasia since it required the animal to be under general anesthesia. This measurement was used to calculate the Lee index, which is equivalent to human BMI. All data collected at any stage of the study were entered into an Excel© spreadsheet. Rat data were also categorized into four groups based on their lifespan, following the comparison of their age to that of humans [17,34,51,52]. These periods were categorized as 1—Teenagers, 2—Young adulthood, 3—Mature adulthood, and 4—Reproductive senescence.

### 4.3. Collection of Biological Material

After a 12 h fasting period (water only), blood glucose levels were obtained using a glucometer by extracting a microdrop of blood from the tip of the awake animals’ tails before they were euthanized with a lethal dose of sodium pentobarbital administered via intraperitoneal injection, to avoid any effect on blood glucose levels. Following euthanasia, blood samples were collected via cardiac puncture under direct visualization for laboratory analysis. The 6 thoracic breasts of each animal, adipose tissue from the subcutaneous region, and perigonadal fat were harvested. A sample of the breast tissue was sent for histological analysis, and another sample was sent for gene expression analysis. During the collection, the volume of fat in the inguinal region and the weight of perigonadal fat were evaluated. The collected and analyzed data are presented in Table 2 and Table 3.

### 4.4. Macroscopic and Histological Analysis of the Breasts

The breast tissue collected from each euthanized animal was initially analyzed macroscopically and then sent to the Laboratory of Special Pathology of the Women’s Hospital, Professor Dr. J. A. Pinotti—UNICAMP. The pathologists responsible for tumor processing and pathological evaluation had no contact with the animals and were not involved in any activities conducted at the animal facility. They received only numbered slides at the pathology laboratory for analysis. Therefore, the evaluations were performed in a blinded manner, without knowledge of the animals’ group allocations. Tissues were immersed in 10% buffered formaldehyde solution and processed for paraffin inclusion. Subsequently, they were cut into 4 μ-thick sections using a microtome, placed on slides, and stained with hematoxylin and eosin (HE) for microscopic analysis.

At least one slide corresponding to each of the thoracic breasts of each rat was examined by a pathologist who was unaware of the origin of the material. The histological analysis followed specific criteria are described as follows (Tables S1 and S2):

1. Number of cell layers in terminal bulbs;
2. Morphology of ductal cells;
3. Evaluation of the presence of Ductal Ectasia and Nuclear Atypia;
4. Evaluation of the presence of a lesion/tumor and characteristics of the lesion.

### 4.5. Analysis of Peripheral Serum Levels

Blood collected by intracardiac puncture in a heparinized tube during tissue extraction was centrifuged at 2400 rpm, 40 °C for 15 min to obtain plasma. Plasma concentrations of insulin, insulin-like growth factor type 1 (IGF-1), C-peptide, leptin, adiponectin, estrone (E1), Leukemia Inhibition Factor (Lif), and C-reactive protein (CRP) were quantified using ELISA (MILLIPLEX, Merck Millipore, Burlington, MA, USA). The results are shown in Table 3.

### 4.6. Immunohistochemical Analysis

The immunohistochemical technique was performed based on histological findings in the Special Pathology Laboratory of the Women’s Hospital Professor, Dr. J. A. Pinotti—UNICAMP, as follows: new sections of paraffin-embedded tissue were placed on histological slides using an organosilane adhesive. After deparaffinization, the sections were hydrated in three water baths, followed by washing under running water for three minutes. To inhibit endogenous peroxidase activity, the slides underwent three three-minute baths each in a 3% hydrogen peroxide solution, followed by another water wash for three minutes. Antigen retrieval was performed by immersing the sections in a specific buffer according to the manufacturer’s guidelines for each kit, at approximately 98 °C for 30 min, using a Pascal pan. After cooling, the slides were washed in running and distilled water and then placed in a phosphate buffer solution (PBS) for five minutes. The slides were incubated with primary antibodies for 30 min in a greenhouse at 37 °C, followed by overnight incubation (16–20 h) at 4 °C in a humid chamber. The next morning, after three five-minute washes in PBS at room temperature, the sections were incubated with a peroxidase-labeled polymer for one hour at 37 °C. Staining was performed using a 60 mg DAB solution (3,3′-diaminobenzidine tetrahydrochloride, Novocastra, code 7169) dissolved in 100 mL of PBS with 1 mL of dimethylsulfoxide and 0.5 mL of 10% hydrogen peroxide, followed by washing under running water. Counter-staining was performed with Harris hematoxylin, followed by another wash in running water. The sections were dehydrated in absolute alcohol baths, passed through xylene, and then mounted with Entellan resin (Merck, code 7961). The positivity of the reaction was determined by membrane, cytoplasm, or nucleus staining, depending on the antibody. The slides were examined under an optical microscope at 400× magnification. The expression of markers in the lesions (benign and malignant) was analyzed, including positivity for estrogen receptor (ER), human epidermal growth factor receptor 2 (HER2), and the cell proliferation index marker Ki-67 (Tables S1 and S2). Immunohistochemical technique was performed using well-validated antibodies and optimized protocols, and included internal negative controls (sections stained without primary antibody) to assess nonspecific staining. Positive controls were used, as shown in Table 6.

### 4.7. Selection and Analysis of Marker Genes for Breast Cancer Development

To identify genes that could serve as markers for pre-cancer or cancer, a PCR Array (Rat Breast Cancer PARN-131ZA—Qiagen^®^, Venlo, The Netherlands) containing 84 target genes was used. Figures and tables were used for data pooling.

RNA was extracted using the PureLinkTM RNA Mini Kit (Invitrogen^®^, Carlsbad, CA, USA), and cDNA was synthesized using 2 μg of total RNA with the High-Capacity cDNA Kit (Applied Biosystems^®^, Waltham, MA, USA). The cDNA was diluted to a concentration of 5 ng/μL, and then 4 μL (20 ng) per plate of the PCR Array was used.

### 4.8. Statistical Analysis

Statistical analysis was conducted using SPSS version 20 for Macintosh (IBM^®^, Armonk, NY, USA). Data distribution was assessed using the Kolmogorov–Smirnov and Shapiro–Wilk tests. Normally distributed data were analyzed using paired *t*-tests, one-way ANOVA, and repeated-measures ANOVA, with Tukey’s post hoc test for multiple comparisons. Results are reported as means ± standard deviation (SD). For non-normally distributed data, the Kruskal–Wallis test was applied, and results are presented as medians with interquartile ranges (IQRs). For gene expression analyses, *p*-values were adjusted for multiple testing using the Benjamini–Hochberg method to control the false discovery rate (FDR). A significance threshold of *p* < 0.05 was used for all statistical tests.

## 5. Conclusions

We developed a rat model that more closely replicates the biological conditions associated with increased breast cancer risk. Despite the limitation of relatively low tumor incidence—although threefold higher in middle-aged, exposed rats than in the controls—this model successfully mapped key metabolic and genomic alterations in both cancerous and non-cancerous mammary glands. Our findings demonstrate the potential of early-life cafeteria diet exposure to drive metabolic dysfunction and create a pro-tumorigenic environment in mammary tissue. Importantly, the model maintains survival rates suitable for long-term preclinical studies, allowing extended observation of tumor development. Moreover, genomic analysis revealed that six months of cafeteria diet exposure, initiated at four weeks of age, was sufficient to induce an approximately 12% incidence of ductal carcinoma, predominantly among younger rats. Future refinements—such as incorporating low-dose carcinogens, ethanol exposure, or altered environmental conditions—could enhance tumor yield while preserving the model’s metabolic and genomic relevance. This approach provides a valuable platform for investigating the mechanistic links between early metabolic dysfunction and breast cancer risk, and may support the development of targeted prevention strategies.

## Figures and Tables

**Table 1:** Murinometric data collected during biological material extraction and characteristics of tumors identified in the Control group.

Rats	Age at Extraction (Weeks of Life)	Phase of Life	Weight	NAL (cm)	Lee Index	FBG	VIF (mL)	WPF (g)	Cause of Death
CB011	16	Very Young	258.20	20.70	0.00	81.00	2.00	3.09	SE
CB012	16		256.87	20.70	0.00	75.00	3.00	6.06	SE
CB013	16		216.68	20.60	0.00	57.00	1.00	1.18	SE
CB021	16		257.72	20.50	0.00	58.00	2.00	2.81	SE
CB023	16		261.24	21.30	0.00	82.00	2.00	3.94	SE
CB031	25		237.70	22.00	0.00	68.00	1.50	3.32	SE
C0B32	25		278.43	21.00	0.00	67.00	1.50	3.18	SE
CB033	25		276.15	22.00	0.00	74.00	1.50	1.88	SE
CA021	26		260.07	21.00	0.00	70.25	1.00	1.45	SE
**Mean**			**255.90**	**21.09**	**0.00**	**70.25**	**1.72**	**2.99**	**NA**
CB051	52	Reproductive Age	343.50	21.60	0.00	66.00	nr	nr	SE
CB043	72		447.00	21.50	0.00	92.00	11.50	24.50	SE
CB052	72		362.30	22.50	0.00	90.00	9.40	9.40	SE
CB053	72		397.40	21.00	0.00	92.00	5.50	12.50	SE
CA022	73		329.40	21.50	0.00	88.00	4.00	7.00	SE
CA032	73		369.90	21.50	0.00	85.00	2.50	5.30	SE
**Mean**			**374.92**	**21.60**	**0.00**	**85.50**	**6.58**	**11.74**	**NA**
CA031	89	Reproductive Senescence	332.60	22.50	0.00	96.00	nr	nr	SE
CA053	89		353.70	21.50	0.00	76.00	nr	nr	SE
CA041	99		275.90	nr	nr	nr	nr	nr	Death
CA011	101		303.70	nr	nr	nr	nr	nr	SE
CA033	102		292.00	21.50	0.00	97.00	2.00	0.90	SE
CA052	102		368.70	20.50	0.00	89.00	9.00	2.60	SE
CB022	102		299.60	21.00	0.00	102.00	5.00	2.00	SE
CB041	102		343.60	20.00	0.00	77.00	10.00	1.70	SE
CB042	101		286.10	20.50	0.00	89.00	3.00	0.00	SE
CA012	103		293.70	nr	nr	nr	nr	nr	SE
CA013	103		333.60	nr	nr	nr	nr	nr	SE
CA023	104		329.70	nr	nr	nr	nr	nr	SE
CA042	104		337.40	nr	nr	nr	nr	nr	SE
CA043	104		308.50	nr	nr	nr	nr	nr	SE
CA051	104		321.40	nr	nr	nr	nr	nr	SE
**Mean**			**318.68**	**21.07**	**0.00**	**89.43**	**5.80**	**1.44**	**NA**

NAL (Naso-anal length), FBG (Fasting Blood Glucose), VIF (Volume of Inguinal Fat), WPF (Weight of Perigonadal Fat), NA (Not Applicable), nr (Not registered), SE (Scheduled euthanasia).

**Table 2:** Murinometric data collected during biological material extraction and characteristics of tumors identified in the Diet group.

Rats	Age at Extraction (Weeks of Life)	Phase of Life	Weight	NAL (cm)	Lee Index	FBG	VIF (mL)	WPF (g)	Cause of Death
DB061	16	Very Young	353.52	22.40	0.00	68.00	8.00	11.19	SE
DB062	16		378.95	22.30	0.00	81.00	12.50	17.60	SE
DB063	16		346.44	22.90	0.00	81.00	7.50	11.92	SE
DB071	16		302.35	21.10	0.00	81.00	5.00	nr	SE
DB073	16		282.02	21.70	0.00	77.00	4.50	6.57	SE
DB081	25		421.73	23.00	0.00	82.00	10.50	13.77	SE
DB082	25		432.59	21.50	0.00	87.00	10.50	18.02	SE
DB083	25		489.13	24.00	0.00	91.00	12.00	17.95	SE
DA071	26		363.58	22.00	0.00	71.00	5.50	15.71	SE
**Mean**			374.48	22.32	0.00	79.89	8.44	14.09	**NA**
DB103	52	Reproductive Age	526.10	22.30	0.00	73.00	nr	nr	SE
DA091	53		368.30	21.50	0.00	77.00	nr	nr	SE
DB091	72		506.30	23.50	0.00	80.00	5.50	20.20	SE
DB101	72		577.30	23.00	0.00	88.00	17.00	33.00	SE
DA063	73		407.60	22.50	0.00	93.00	3.50	24.00	SE
DA072	73		508.10	23.50	0.00	98.00	14.00	45.70	SE
**Mean**			482.28	22.72	0.00	84.83	10.00	30.73	**NA**
DA082	83	Reproductive Senescence	299.30	21.00	0.00	nr	nr	nr	ES
DA092	84		361.10	22.00	0.00	nr	nr	nr	Death
DA101	89		440.80	23.00	0.00	nr	nr	nr	Death
DA081	89		464.60	22.50	0.00	90.00	nr	nr	SE
DB102	92		288.80	nr	nr	nr	nr	nr	Death
DA062	94		258.40	nr	nr	nr	nr	nr	Death
DA093	94		491.70	nr	nr	nr	nr	nr	Death
DA102	94		315.90	nr	nr	nr	nr	nr	Death
DA061	95		322.40	nr	nr	nr	nr	nr	Death
DA073	96		456.90	nr	nr	nr	nr	nr	Death
DB072	99		341.30	22.50	0.00	nr	nr	nr	ES
DB092	101		472.50	22.40	0.00	84.00	14.00	56.90	SE
DB093	101		405.30	21.00	0.00	111.00	14.00	66.00	SE
DA103	102		319.50	21.50	0.00	93.00	7.00	3.80	SE
DA083	102		493.10	20.00	0.00	81.00	19.00	21.70	SE
**Mean**			**382.11**	**21.77**	**0.00**	**91.80**	**13.50**	**37.10**	**NA**
***p* (Control vs. Cafeteria Diet)**			0.011 *	0.352	0.396	0.362	0.022 *	0.046 *	<0.001 *

NAL (Naso-anal length), FBG (Fasting Blood Glucose), VIF (Volume of Inguinal Fat), WPF (Weight of Perigonadal Fat), NA (Not Applicable), nr (Not registered). SE (Scheduled euthanasia), ES (Euthanasia due to animal suffering). * indicates statistical significance of the values.

**Table 3:** Plasma concentrations of insulin, insulin-like growth factor type 1 (IGF-1), C-peptide, leptin, adiponectin, estrone (E1), leukemia inhibitory factor (Lif), and C-reactive protein (CRP) in the fasting state, quantified by ELISA.

					CI95%					
	N	Mean	SD	SE	Lower Limit	Upper Limit	Min.	Max.	Sig.	*p*
Adiponectin, μg/mL	Control	14	19.906	9.553	2.553	14.39	25.421	8.79	45.94	0.106
	Diet	13	26.998	12.332	3.42	19.546	34.451	2.99	42.89	
IGF-1, ng/mL	Control	14	140.521	137.249	36.681	61.276	219.766	34.64	518.71	0.838
	Diet	14	149.388	83.643	22.355	101.094	197.682	37.91	316.01	
LIF (Leukemia inhibitory factor), pg/mL	Control	5	106.494	148.817	66.553	−78.287	291.275	9.85	370.43	0.504
	Diet	5	58.442	37.697	16.858	11.636	105.248	15.25	105.82	
Leptin *, ng/mL	Control	14	2.135	3.165	0.846	0.308	3.962	0.13	12.69	<0.001 *
	Diet	13	8.487	4.292	1.191	5.893	11.081	3.18	15.73	
Peptide C, ng/mL	Control	13	49.419	78.426	21.751	2.026	96.811	0.775	288.001	0.8
	Diet	13	43.245	37.654	10.443	20.491	65.999	1.55	124.455	
Insulin, ng/ml	Control	3	305.477	423.488	244.501	−746.526	1357.48	54.44	794.42	0.19
	Diet	6	70.005	46.288	18.897	21.428	118.582	2.31	124.32	
Estrone (E1), pg/mL	Control	7	0.657	0.205	0.077	0.468	0.846	0.302	0.884	0.944
	Diet	9	0.666	0.27	0.09	0.458	0.873	0.242	1.031	
CRP (C-Reactive Protein), μg/mL	Control	4	386.97	402.703	201.351	−253.82	1027.76	39.46	876.46	0.562
	Diet	9	518.339	351.357	117.119	248.262	788.416	27.37	1090.14	

* indicates statistical significance of the values.

**Table 4:** Assays used in qPCR. Table with the name of the genes, the assay code, and the nucleotide region used to construct the primers. The assays were purchased from IDT DNA Technologies or Thermo Fischer Scientific. NR3C1 (“nuclear receptor subfamily 3, group C, member 1”); BRCA2 (“breast cancer 2”); KRT5 (“Keratin 5”); HIF-1-alpha (“Hypoxia-inducible factor 1-alpha”); ID-1 (“DNA-binding protein inhibitor ID-1”); MMP-1 (“matrix metalloproteinase-2”); MUC1 (“Mucin 1, cell surface associated”); THBS1 (“Thrombospondin 1”); TIMP1 (“TIMP metallopeptidase inhibitor 1”); TWIST1 (“Twist-related protein 1”); COX-2 (“Ciclooxigenase-2”); PAI-1 (“Plasminogen activator inhibitor-1”); VEGF-A (“Vascular endothelial growth factor A”); TGF-beta1 (“Transforming growth factor beta 1”).

Gene Name	Assay IDT DNA Technologies	Ref Seq	Exon Boundary
*Brca2*	Rn.PT.58.7981753	NM_031542(1)	7–9
*Hif-1ɑ*	Rn.PT.58.12503723	NM_024359(1)	5–6
*Id-1*	Rn.PT.58.37482699.g	NM_012797(1)	1–2
*Krt5*	Rn.PT.58.45199866	NM_183333(1)	6–8
*Mmp2*	Rn.PT.58.8937436	NM_031054(1)	10–11
*Muc1*	Rn.PT.58.45226306	NM_012602(1)	6–7
*Nr3c1*	Rn.PT.58.35361161	NM_012576(1)	7–8
*Thbs1*	Rn.PT.58.44657050	NM_001013062(1)	5–6
*Timp-1*	Rn.PT.58.23885446	NM_053819(1)	2–4
*Twist-1*	Rn.PT.58.10199852.gs	NM_053530(1)	1–2
*Vegf-ɑ*	Rn.PT.58.34830017	NM_001110334(1)	5c-9
**Gene Name**	**Assay Thermo Fisher**	**Ref Seq**	**Exon Boundary**
*β-actin*	Rn00667869_m1	NM_031144.3	4–5
*Cox-2*	Rn01483830_g1	NM_017232.3	9–10
*Gapdh*	Rn99999916_s1	NM_017008.4	3–3
*Tgfβ-1*	Rn00572010_m1	NM_021578.2	1–2
*Pai-1*	Rn01481341_m1	NM_012620.1	7–8

**Table 5:** Composition of the cafeteria diet.

	Energy Value kj/100 g	Carbohydrate g/100 g	Protein g/100 g	Total Fat g/100 g	Sodium g/100 g
Cheese Snack Ball (Pepsico, Brazil)	1948	72	6.4	17.2	676
Bacon Flavored Snack Troféu (Santa Helena, Brazil)	2200	56	8.8	30	1040
Wheatflour Biscuit (Zadimel, Brazil)	1793	73	8	10.7	300
Chocolate cake (Nutrella, Brazil)	1798	55	5	21.7	141.7
Coca- cola (Coca-cola, Brazil)	178	11	0	0	5
Guaraná Soda (Antartica, Brazil)	168	10	0	0	5.5
Italian Salame (Sadia, Brazil)	1822	2	22	38	1140
Mixed Sausage (Sadia, Brazil)	1554	1.4	16	34	1342
Bread (Nutrella, Brazil)	1328	54	11.2	6.2	300
Chocolate Waffer (Bauducco, Brazil)	2176	63	5	27	113
Mortadella (Frimesa, Brazil)	845	2	12	16	1545
Marshmallow (Fini, Brazil)	1423	80	5	0	46

**Table 6:** Detailed antibodies used in immunohistochemical analysis.

Antibody	Model	Brand	Catalog Number	Dilution Ratio	Control
Estrogen receptor (ER)	1D5	Invitrogen (Carlsbad, CA, USA) Thermofisher (Waltham, MA, USA)	MA5-13191	1:50	Normal mammary epithelia
Progesterone receptor (PR)	Alpha PR6	Invitrogen Thermofisher	MA1-411	1:150	Normal mammary epithelia
HER2/C-ERB B2	policlonal	Dako (Glostrup, Denmark)	A0485	1:200	Sebaceous gland
Ki67	SP6	Roche (Basel, Switzerland)	M3060	Pure	Epidermic basal layer
PAI-1	policlonal	Abcam (Cambridge, UK)	ab66705	1:100	Human placenta

## Data Availability

The data supporting the reported results can be obtained by emailing Francisco Claro, Jr. at fclarojr@gmail.com.

## Supplementary Materials

The following supporting information can be downloaded at: https://www.mdpi.com/article/10.3390/ijms26157296/s1.
