# Supplementary material for: Gene Monitoring in Obesity-Induced Metabolic Dysfunction in Rats: Preclinical Data on Breast Neoplasia Initiation
Source: Int J Mol Sci. 2025 Jul 28;26(15):7296. doi: 10.3390/ijms26157296 (PMC12346978; doi:10.3390/ijms26157296)
Supplement: Supplementary file 1 [file ijms-26-07296-s001.zip › ijms-3728989-supplementary.pdf]

Supplementary Materials

Table S1. Histological analysis (HE, 400X) of mammary glands in the Control group rats.

Rat	Breast/s	Age (weeks)	NC TEB (n)	MDC	DE	NA	Tumor	ER	CERB-B2	Ki67 (%)	PAI-1
CA21	CA 21 MA	26	1	CE	N	N	N	N/A	N/A	N/A	N/A
	CA 21 MB/MC		1	FE/CE	N	N	N	N/A	N/A	N/A	N/A
	CA 21 MD		1	FE/CE	N	N	N	N/A	N/A	N/A	N/A
	CA 21 ME		2	CE	N	N	N	N/A	N/A	N/A	N/A
	CA 21 MF		2	CE	N	N	N	N/A	N/A	N/A	N/A
CA22	CA 22 MA	73	2	FE/CE	DE	N	cysts, fibroadenoma	N/A	N/A	N/A	N/A
	CA 22 MB/MC		2	FE/CE	DE	N	N	N/A	N/A	N/A	N/A
	CA 22 MD		2	FE/CE	DE	N	cysts with apocrine metaplasia	N/A	N/A	N/A	N/A
	CA 22 ME		4	FE/CE	DE	NA	cysts, fibroadenoma	N/A	N/A	N/A	N/A
	CA 22 MF		2	CC/FE/CE	DE	N	cysts with calcification, fibrioadenoma	N/A	N/A	N/A	N/A
CA31	CA 31 MA	84	2	CC/CE	N	N	N	N/A	N/A	N/A	N/A
	CA 31 MB		1	CE	N	N	N	N/A	N/A	N/A	N/A
	CA 31 MC		1	CE	N	N	N	N/A	N/A	N/A	N/A
	CA 31 MD		3	CE	DE	N	N	N/A	N/A	N/A	N/A
	CA 31 ME		1	CE	N	N	N	N/A	N/A	N/A	N/A
	CA 31 MF		10	CE	N	NA	ductal carcinoma (FT3, GN2, IM1)	5	1+	70	SP
CA32	CA 32 MA	73	2	CC/FE/CE	N	N	cysts	N/A	N/A	N/A	N/A
	CA 32 MB		2	CC/FE/CE	DE	NA	cysts, intraductal microcalcifications, ductal carcinoma (FT3, CN2, IM1) colonizing fibroadenoma	3	1+	<1	SP
	CA 32 MC		2	CE	DE	N	N	N/A	N/A	N/A	N/A
	CA 32 MD		2	CE	DE	N	cysts	N/A	N/A	N/A	N/A
	CA 32 ME		2	CC/FE/CE	DE	N	cysts with calcification	N/A	N/A	N/A	N/A
	CA 32 MF		2	CE	DE	NA	ductal carcinoma (FT3, CN2, IM1) colonizing fibroadenoma	4	0	80	SP
CA33	CA 33 MA	96	1	FE/CE	N	N	N	N/A	N/A	N/A	N/A
	CA 33 MB		1	CE	N	N	N	N/A	N/A	N/A	N/A
	CA 33 MC		1	FE/CE	DE	N	N	N/A	N/A	N/A	N/A
	CA 33 MD		1	FE/CE	DE	N	N	N/A	N/A	N/A	N/A
	CA 33 ME		1	FE/CE	N	N	N	N/A	N/A	N/A	N/A
	CA 33 MF		1	FE/CE	DE	N	N	N/A	N/A	N/A	N/A
CA52	CA 52 MA	96	1	FE/CE	N	N	N	N/A	N/A	N/A	N/A
	CA 52 MB		1	CE	N	N	N	N/A	N/A	N/A	N/A
	CA 52 MC		1	CE	N	N	N	N/A	N/A	N/A	N/A
	CA 52 MD		1	CE	N	N	N	N/A	N/A	N/A	N/A
	CA 52 ME		1	FE/CE	N	N	N	N/A	N/A	N/A	N/A
	CA 52 MF		1	CE	N	N	N	N/A	N/A	N/A	N/A
CA53	CA 53 MA	84	-	CE	N	NA	ductal carcinoma (FT3, CN2, IM1)	2	0	60	SP
	CA 53 MB		2	CE	N	N	N	N/A	N/A	N/A	N/A
	CA 53 MC		1	CE	N	N	N	N/A	N/A	N/A	N/A
	CA 53 MD		1	CE	N	N	N	N/A	N/A	N/A	N/A
	CA 53 ME		2	FE/CE	N	N	N	N/A	N/A	N/A	N/A
	CA 53 MF		1	CE	N	N	N	N/A	N/A	N/A	N/A
CB11	CB 11 MA	16	1	CE	N	N	N	N/A	N/A	N/A	N/A
	CB 11 MB		2	CE	N	N	N	N/A	N/A	N/A	N/A
	CB 11 MD		1	CE	N	N	N	N/A	N/A	N/A	N/A
	CB 11 ME		1	CE	N	N	N	N/A	N/A	N/A	N/A
	CB 11 MF		1	CE	N	N	N	N/A	N/A	N/A	N/A
CB12	CB 12 MA	16	1	CE	N	N	N	N/A	N/A	N/A	N/A
	CB 12 MB		1	CE	N	N	N	N/A	N/A	N/A	N/A
	CB 12 MC		1	CE	N	N	N	N/A	N/A	N/A	N/A
	CB 12 MD		1	CE	N	N	N	N/A	N/A	N/A	N/A
	CB 12 MD		1	CE	N	N	N	N/A	N/A	N/A	N/A
	CB 12 ME		1	CE	N	N	N	N/A	N/A	N/A	N/A
	CB 12 MF		1	CE	N	N	N	N/A	N/A	N/A	N/A
CB13	CB 13 MA	16	1	CE	N	N	N	N/A	N/A	N/A	N/A
	CB 13 MB		1	CE	N	N	N	N/A	N/A	N/A	N/A
	CB 13 MC		1	CE	N	N	N	N/A	N/A	N/A	N/A
	CB 13 ME		1	CE	N	N	N	N/A	N/A	N/A	N/A
CB21	CB 21 MA	16	1	FE/CE	N	N	N	N/A	N/A	N/A	N/A
	CB 21 MB		2	CE	N	N	N	N/A	N/A	N/A	N/A
	CB 21 MD		2	FE/CE	DE	N	N	N/A	N/A	N/A	N/A
	CB 21 ME		1	CE	N	N	N	N/A	N/A	N/A	N/A
	CB 21 MF		1	FE/CE	N	N	N	N/A	N/A	N/A	N/A
CB23	CB 23 MB	16	2	CE	DE	N	N	N/A	N/A	N/A	N/A
	CB 23 MC		1	CE	N	N	N	N/A	N/A	N/A	N/A
	CB 23 MD		1	CE	N	N	N	N/A	N/A	N/A	N/A
	CB 23 ME		2	CE	N	N	N	N/A	N/A	N/A	N/A
	CB 23 MF		2	CE	N	N	N	N/A	N/A	N/A	N/A
CB31	CB 31 MA	25	2	CE	N	N	N	N/A	N/A	N/A	N/A
	CB 31 MB		2	CE	N	N	N	N/A	N/A	N/A	N/A
	CB 31 MC		1	CE	N	N	N	N/A	N/A	N/A	N/A
	CB 31 MD		1	CE	N	N	N	N/A	N/A	N/A	N/A
	CB 31 ME		3	CE	DE	N	N	N/A	N/A	N/A	N/A
	CB 31 MF		2	CC/FE/CE	DE	N	N	N/A	N/A	N/A	N/A
CB32	CB 32 MA	25	2	CE	N	N	N	N/A	N/A	N/A	N/A
	CB 32 MB		2	CE	N	N	N	N/A	N/A	N/A	N/A
	CB 32 MC		2	CE	N	N	N	N/A	N/A	N/A	N/A
	CB 32 MD		2	CE	N	N	N	N/A	N/A	N/A	N/A
	CB 32 ME		2	CC/FE/CE	DE	N	N	N/A	N/A	N/A	N/A
	CB 32 MF		1	CC/FE/CE	N	N	N	N/A	N/A	N/A	N/A
CB33	CB 33 MA	25	2	CE	N	N	N	N/A	N/A	N/A	N/A
	CB 33 MB		3	CE	N	N	N	N/A	N/A	N/A	N/A
	CB 33 MC		2	FE/CE	N	N	N	N/A	N/A	N/A	N/A
	CB 33 MD		1	CE	N	N	N	N/A	N/A	N/A	N/A
	CB 33 ME		2	CC/FE/CE	N	N	N	N/A	N/A	N/A	N/A
	CB 33 MF		2	CE	N	N	N	N/A	N/A	N/A	N/A
CB41	CB 41 MA	95	1	CE	N	N	N	N/A	N/A	N/A	N/A
	CB 41 MB		1	CE	N	N	N	N/A	N/A	N/A	N/A
	CB 41 MC		1	CE	N	N	N	N/A	N/A	N/A	N/A
	CB 41 MD		1	CE	N	N	N	N/A	N/A	N/A	N/A
	CB 41 ME		2	CE	N	N	N	N/A	N/A	N/A	N/A
	CB 41 MF		1	CE	N	N	N	N/A	N/A	N/A	N/A
CB42	CB 42 MA	95	1	CE	N	N	N	N/A	N/A	N/A	N/A
	CB 42 MB		1	FE/CE	N	N	N	N/A	N/A	N/A	N/A
	CB 42 MC		1	CE	N	N	N	N/A	N/A	N/A	N/A
	CB 42 MD		1	CE	N	N	cysts	N/A	N/A	N/A	N/A
	CB 42 ME		1	FE/CE	N	N	N	N/A	N/A	N/A	N/A
	CB 42 MF		1	CE	N	N	cysts	N/A	N/A	N/A	N/A
CB51	CB 51 MA	52	2	CE	N	N	N	N/A	N/A	N/A	N/A
	CB 51 MB		2	CE	N	N	N	N/A	N/A	N/A	N/A
	CB 51 MC		2	FE/CE	N	N	N	N/A	N/A	N/A	N/A
	CB 51 MD		2	FE/CE	N	N	N	N/A	N/A	N/A	N/A
	CB 51 ME		2	CE	N	N	N	N/A	N/A	N/A	N/A
	CB 51 MF		1	FE/CE	DE	N	N	N/A	N/A	N/A	N/A

NC TEB = Number of Cell Layers in Terminal Bulbs. MDC = Morphology of Ductal Cells (Flat Epithelium - FE, Cuboidal Epithelium - CE, or modifications for Columnar Cells - CC). DE = Ductal Ectasia (N=No). NA = Nuclear Atypia (N=No). Immunohistochemical staining for ER=Estrogen receptor, CERB-B2, Ki67 (%) and PAI-1. N/A=Not Applicable. SP=Strong Positive

Table S2. Histological analysis (HE, 400X) of mammary glands in the Cafeteria Diet group rats.

Rat	Breast/s	Age (weeks)	NC TEB (n)	MDC	DE	NA	Tumor	ER	CERB-B2	Ki67 (%)	PAI-1
DA61	DA 61 MA	95	1	CE	N	N	N	N/A	N/A	N/A	N/A
	DA 61 MB/MC		1	CE	N	N	N	N/A	N/A	N/A	N/A
	DA 61 MD		1	CE	N	N	fibroadenoma	N/A	N/A	N/A	N/A
	DA 61 ME/MF		1	CE	N	N	N	N/A	N/A	N/A	N/A
DA62	DA 62 MA	94	1	CE	N	N	N	N/A	N/A	N/A	N/A
	DA 62 MB/MC		1	CE	N	N	N	N/A	N/A	N/A	N/A
	DA 62 MD		1	FE/CE	N	N	N	N/A	N/A	N/A	N/A
	DA 62 ME/MF		1	CE	N	N	N	N/A	N/A	N/A	N/A
DA 63	DA 63 MB	73	2	CE	N	N	N	N/A	N/A	N/A	N/A
	DA 63 MC		2	CE	N	N	N	N/A	N/A	N/A	N/A
	DA 63 MD/MF		2	CE	N	NA	ductal carcinoma (FT3, CN2, IM1) colonizing fibroadenoma	2	1+	1	SP
	DA 63 ME		1	CE	N	N	N	N/A	N/A	N/A	N/A
DA71	DA 71 MA	26	1	CE	N	N	N	N/A	N/A	N/A	N/A
	DA 71 MB/MC		2	FE/CE	N	N	N	N/A	N/A	N/A	N/A
	DA 71 MD		1	CE	N	N	N	N/A	N/A	N/A	N/A
	DA 71 ME		1	FE/CE	N	N	fibroadenoma	N/A	N/A	N/A	N/A
	DA 71 MF		1	FE/CE	N	N	N	N/A	N/A	N/A	N/A
DA72	DA 72 MA	73	1	FE/CE	N	N	N	N/A	N/A	N/A	N/A
	DA 72 MB		1	FE/CE	DE	N	N	N/A	N/A	N/A	N/A
	DA 72 MC		1	FE/CE	N	N	N	N/A	N/A	N/A	N/A
	DA 72 MD		1	CE	N	N	fibroadenoma	N/A	N/A	N/A	N/A
	DA 72 ME		1	FE/CE	DE	N	N	N/A	N/A	N/A	N/A
	DA 72 MF		1	CE	N	N	N	N/A	N/A	N/A	N/A
DA 73	DA 73 MA	96	1	FE/CE	N	N	N	N/A	N/A	N/A	N/A
	DA 73 MB		1	FE/CE	DE	N	N	N/A	N/A	N/A	N/A
	DA 73 MC		1	FE/CE	DE	N	Papiloma intraductal	N/A	N/A	N/A	N/A
	DA 73 MD		1	FE/CE	N	N	N	N/A	N/A	N/A	N/A
	DA 73 ME/MF		1	FE/CE	DE	N	N	N/A	N/A	N/A	N/A
DA81	DA 81 MA	89	2	FE/CE	N	N	N	N/A	N/A	N/A	N/A
	DA 81 MB/MC		1	CE	N	N	N	N/A	N/A	N/A	N/A
	DA 81 MD/ME		1	CE	N	N	N	N/A	N/A	N/A	N/A
	DA 81 MF		1	FE/CE	N	N	fibroadenoma	N/A	N/A	N/A	N/A
DA82	DA 82 MA	83	2	CE	N	N	N	N/A	N/A	N/A	N/A
	DA 82 MB/MC		2	CE	N	N	N	N/A	N/A	N/A	N/A
	DA 82 MD		2	CE	N	N	N	N/A	N/A	N/A	N/A
	DA 82 ME		1	CE	DE	N	N	N/A	N/A	N/A	N/A
	DA 82 MF		2	CE	N	N	N	N/A	N/A	N/A	N/A
DA83	DA 83 MA	25	2	FE/CE	DE	N	intraductal papilloma	N/A	N/A	N/A	N/A
	DA 83 MB		1	FE/CE	N	N	N	N/A	N/A	N/A	N/A
	DA 83 MC		1	CE	N	N	N	N/A	N/A	N/A	N/A
	DA 83 MD		1	FE/CE	DE	N	N	N/A	N/A	N/A	N/A
	DA 83 ME		1	FE/CE	DE	N	N	N/A	N/A	N/A	N/A
	DA 83 MF		1	FE/CE	DE	N	N	N/A	N/A	N/A	N/A
DA91	DA 91 MA	53	2	FE/CE	N	N	N	N/A	N/A	N/A	N/A
	DA 91 MB		2	FE/CE	N	N	cyst	N/A	N/A	N/A	N/A
	DA 91 MC		2	CE	N	N	N	N/A	N/A	N/A	N/A
	DA 91 MD		2	FE/CE	N	N	N	N/A	N/A	N/A	N/A
	DA 91 ME/MF		2	FE/CE	N	N	N	N/A	N/A	N/A	N/A
DA92	DA 92 MA	84	1	CE	N	N	N	N/A	N/A	N/A	N/A
	DA 92 MB		2	CE	N	N	N	N/A	N/A	N/A	N/A
	DA 92 MC		2	CE	N	N	N	N/A	N/A	N/A	N/A
	DA 92 ME		2	CE	N	N	N	N/A	N/A	N/A	N/A
	DA 92 MD/MF		2	CE	N	N	N	N/A	N/A	N/A	N/A
DA93	DA 93 MA	94	1	FE/CE	N	N	N	N/A	N/A	N/A	N/A
	DA 93 MB/MC		1	CE	N	N	N	N/A	N/A	N/A	N/A
	DA 93 MD		1	FE/CE	N	N	N	N/A	N/A	N/A	N/A
	DA 93 ME/MF		1	CE	N	N	N	N/A	N/A	N/A	N/A
DB101	DB 101 MA	72	2	FE/CE	N	N	N	N/A	N/A	N/A	N/A
	DB 101 MB		2	CE	N	N	N	N/A	N/A	N/A	N/A
	DB 101 MC		2	CE	N	N	N	N/A	N/A	N/A	N/A
	DB 101 MD		1	CE	N	N	N	N/A	N/A	N/A	N/A
	DB 101 ME		2	CE	N	N	N	N/A	N/A	N/A	N/A
	DB 101 MF		2	CE	N	NA	ductal carcinoma (FT3, CN2, IM1) colonizing fibroadenoma	2	1+	<1	MP
DB102	DB 102 MA	92	1	CE	N	N	N	N/A	N/A	N/A	N/A
	DB 102 MB		2	CE	N	N	N	N/A	N/A	N/A	N/A
	DB 102 MC		1	FE/CE	DE	N	N	N/A	N/A	N/A	N/A
	DB 102 MD		1	CE	N	N	N	N/A	N/A	N/A	N/A
	DB 102 ME		2	CE	N	N	N	N/A	N/A	N/A	N/A
	DB 102 MF		1	CE	N	N	N	N/A	N/A	N/A	N/A
DB103	DB 102 MA	52	2	CE	N	N	N	N/A	N/A	N/A	N/A
	DB102 MB/MC		2	CE	N	N	N	N/A	N/A	N/A	N/A
	DB102 MD		2	CE	N	N	cyst	N/A	N/A	N/A	N/A
	DB102 MB/ME		2	CE	N	N	N	N/A	N/A	N/A	N/A
DB61	DB 61 MA	16	1	CE	N	N	N	N/A	N/A	N/A	N/A
	DB 61 MB		1	FE/CE	DE	N	N	N/A	N/A	N/A	N/A
	DB 61 MC		2	FE/CE	DE	N	N	N/A	N/A	N/A	N/A
	DB 61 MD		1	FE/CE	N	N	N	N/A	N/A	N/A	N/A
	DB 61 ME		1	FE/CE	N	N	N	N/A	N/A	N/A	N/A
	DB 61 MF		1	CE	N	N	N	N/A	N/A	N/A	N/A
DB62	DB 62 MA	16	2	CE	N	N	N	N/A	N/A	N/A	N/A
	DB 62 MB		2	FE/CE	DE	N	N	N/A	N/A	N/A	N/A
	DB 62 MC		2	FE/CE	N	N	N	N/A	N/A	N/A	N/A
	DB 62 MD		2	CE	N	N	N	N/A	N/A	N/A	N/A
	DB 62 ME		1	FE/CE	N	N	N	N/A	N/A	N/A	N/A
	DB 62 MF		1	CE	N	N	N	N/A	N/A	N/A	N/A
DB63	DB 63 MA	16	1	FE/CE	N	N	N	N/A	N/A	N/A	N/A
	DB 63 MB		2	CE	DE	N	N	N/A	N/A	N/A	N/A
	DB 63 MC		2	FE/CE	DE	N	N	N/A	N/A	N/A	N/A
	DB 63 MD		1	FE/CE	N	N	N	N/A	N/A	N/A	N/A
	DB 63 ME		1	FE/CE	N	N	N	N/A	N/A	N/A	N/A
	DB 63 MF		1	CE	N	N	N	N/A	N/A	N/A	N/A
DB71	DB 71 MA	16	1	CE	N	N	N	N/A	N/A	N/A	N/A
	DB 71 MB		1	CE	N	N	N	N/A	N/A	N/A	N/A
	DB 71 MC		1	CE	N	N	N	N/A	N/A	N/A	N/A
	DB 71 MD		1	CE	N	N	N	N/A	N/A	N/A	N/A
	DB 71 ME		1	FE/CE	N	N	N	N/A	N/A	N/A	N/A
	DB 71 MF		1	CE	N	N	N	N/A	N/A	N/A	N/A
DB72	DB 72 MA	99	1	FE/CE	N	N	N	N/A	N/A	N/A	N/A
	DB 72 MB		1	FE/CE	DE	N	N	N/A	N/A	N/A	N/A
	DB 72 MC		1	FE/CE	N	N	N	N/A	N/A	N/A	N/A
	DB 72 MD		1	FE/CE	N	N	N	N/A	N/A	N/A	N/A
	DB 72 ME/MF		1	FE/CE	N	N	N	N/A	N/A	N/A	N/A
DB73	DB 73 MA	16	1	CE	N	N	N	N/A	N/A	N/A	N/A
	DB 73 MB		2	CE	N	N	N	N/A	N/A	N/A	N/A
	DB 73 MC		1	CE	N	N	N	N/A	N/A	N/A	N/A
	DB 73 MD		1	CE	N	N	N	N/A	N/A	N/A	N/A
	DB 73 ME		1	CE	DE	N	N	N/A	N/A	N/A	N/A
	DB 73 MF		1	CE	N	N	N	N/A	N/A	N/A	N/A
DB81	DB 81 MA	25	2	FE/CE	DE	N	N	N/A	N/A	N/A	N/A
	DB 81 MC		1	CE	N	N	N	N/A	N/A	N/A	N/A
	DB 81 MD		1	CE	N	N	N	N/A	N/A	N/A	N/A
	DB 81 ME		3	CE	DE	N	N	N/A	N/A	N/A	N/A
	DB 81 MF		2	CE	N	N	N	N/A	N/A	N/A	N/A
DB82	DB 82 MA	25	2	FE/CE	DE	N	N	N/A	N/A	N/A	N/A
	DB 82 MB		2	CE	N	N	N	N/A	N/A	N/A	N/A
	DB 82 MC		2	CE	DE	N	N	N/A	N/A	N/A	N/A
	DB 82 MD		1	CE	N	N	N	N/A	N/A	N/A	N/A
	DB 82 ME		2	FE/CE	N	N	fibroadenoma	N/A	N/A	N/A	N/A
	DB 82 MF		2	FE/CE	N	N	N	N/A	N/A	N/A	N/A
DB83	DB 83 MA	25	1	CE	N	N	N	N/A	N/A	N/A	N/A
	DB 83 MB		1	CE	N	N	N	N/A	N/A	N/A	N/A
	DB 83 MC		1	CE	N	N	N	N/A	N/A	N/A	N/A
	DB 83 MD		2	CE	N	N	N	N/A	N/A	N/A	N/A
	DB 83 ME		1	FE/CE	DE	N	N	N/A	N/A	N/A	N/A
	DB 83 MF		1	CE	N	N	N	N/A	N/A	N/A	N/A
DB91	DB 91 MA	72	2	CE	N	N	N	N/A	N/A	N/A	N/A
	DB 91 MB		2	CE	N	N	N	N/A	N/A	N/A	N/A
	DB 91 MC		2	CE	N	NA	fibrosis and foci of ductal carcinoma (FT3, CN2, IM1)	3	0	<1	SP
	DB 91 MD		2	CE	N	N	N	N/A	N/A	N/A	N/A
	DB 91 ME		2	CE	N	N	N	N/A	N/A	N/A	N/A
DB92	DB 92 MA	101	1	FE/CE	N	N	N	N/A	N/A	N/A	N/A
	DB 92 MB		1	FE/CE	N	N	N	N/A	N/A	N/A	N/A
	DB 92 MC		1	FE/CE	N	N	N	N/A	N/A	N/A	N/A
	DB 92 MD		1	FE/CE	DE	N	N	N/A	N/A	N/A	N/A
	DB 92 ME		1	FE/CE	DE	N	N	N/A	N/A	N/A	N/A
	DB 92 MF		1	FE/CE	DE	N	N	N/A	N/A	N/A	N/A
DB93	DB 93 MA	101	1	FE/CE	DE	N	N	N/A	N/A	N/A	N/A
	DB 93 MB		1	FE/CE	N	N	N	N/A	N/A	N/A	N/A
	DB 93 MC		2	FE/CE	N	N	fibroadenoma	N/A	N/A	N/A	N/A
	DB 93 MD		1	FE/CE	N	N	N	N/A	N/A	N/A	N/A
	DB 93 ME		1	CE	N	N	N	N/A	N/A	N/A	N/A
	DB 93 MF		1	CE	N	N	fibroadenoma	N/A	N/A	N/A	N/A

NC TEB = Number of Cell Layers in Terminal Bulbs. MDC = Morphology of Ductal Cells (Flat Epithelium - FE, Cuboidal Epithelium - CE, or modifications for Columnar Cells - CC). DE = Ductal Ectasia (N=No). NA = Nuclear Atypia (N=No). Immunohistochemical staining for ER=Estrogen receptor, CERB-B2, Ki67 (%) and PAI-1. N/A=Not Applicable. SP=Strong Positive. MP=moderate positive
